# Alginate-Chitosan Biodegradable and Biocompatible Based Hydrogel for Breast Cancer Immunotherapy and Diagnosis: A Comprehensive Review

**DOI:** 10.1021/acsabm.3c00984

**Published:** 2024-05-24

**Authors:** Pratikshya Patra, Tarun Kumar Upadhyay, Nawaf Alshammari, Mohd Saeed, Kavindra Kumar Kesari

**Affiliations:** † Department of Biotechnology, Parul Institute of Applied Sciences and Animal Cell Culture and Immunobiochemistry Lab, Research and Development Cell, Parul University, Vadodara, Gujarat 391760, India; ‡ Department of Biology, College of Science, University of Hail, Hail 53962, Saudi Arabia; § Department of Applied Physics, School of Science, Aalto University, Espoo FI-00076, Finland; ∥ Centre of Research Impact and Outcome, Chitkara University, Rajpura 140417, Punjab, India

**Keywords:** Breast cancer, Immunotherapy, Hydrogel, Chitosan, Alginate

## Abstract

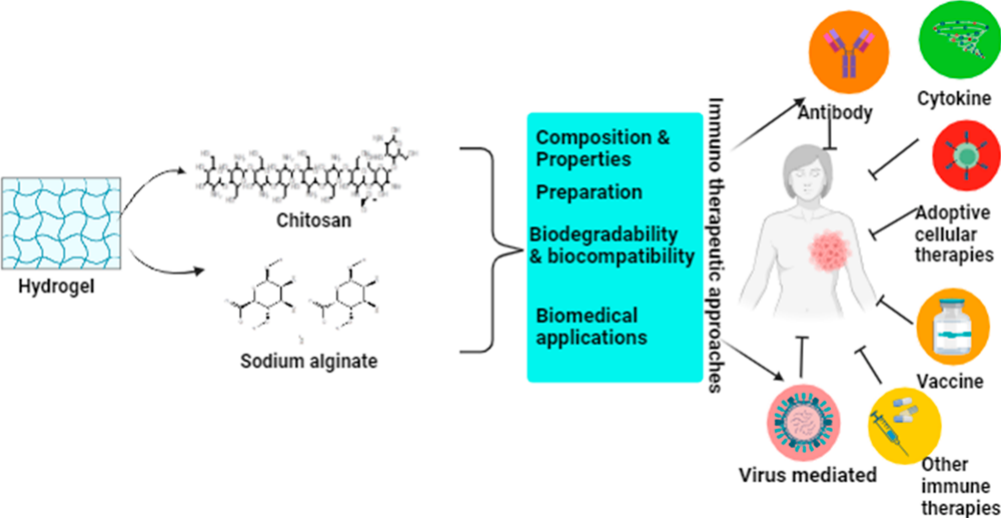

Breast cancer is the most common type of cancer and the second leading cause of cancer-related mortality in females. There are many side effects due to chemotherapy and traditional surgery, like fatigue, loss of appetite, skin irritation, and drug resistance to cancer cells. Immunotherapy has become a hopeful approach toward cancer treatment, generating long-lasting immune responses in malignant tumor patients. Recently, hydrogel has received more attention toward cancer therapy due to its specific characteristics, such as decreased toxicity, fewer side effects, and better biocompatibility drug delivery to the particular tumor location. Researchers globally reported various investigations on hydrogel research for tumor diagnosis. The hydrogel-based multilayer platform with controlled nanostructure has received more attention for its antitumor effect. Chitosan and alginate play a leading role in the formation of the cross-link in a hydrogel. Also, they help in the stability of the hydrogel. This review discusses the properties, preparation, biocompatibility, and bioavailability of various research and clinical approaches of the multipolymer hydrogel made of alginate and chitosan for breast cancer treatment. With a focus on cases of breast cancer and the recovery rate, there is a need to find out the role of hydrogel in drug delivery for breast cancer treatment.

## 1 Introduction

Cancer is one of the world’s major health problems and is a burden of disease globally. It accuse for motility, morbidity, and the primary cause of death among six global deaths.^1^ Globally, there are more than 30 different cancers present. Breast cancer (BC) is one of the most prevalent and aggressive types of cancer, primarily affecting women. It overtakes lung cancer and is now reported as the leading cause of cancer in 2020. It is predicted that there will be 23 million new cases, which will be 11.7% of the total cancers.^2^ Despite it has been been reported recently a tremendous improvements in BC detection, treatment, and diagnosis over the last decades, even though it has found a significant global health burden.^3^ The metastasis nature of cancer is the most critical issue of clinical treatment.^4^ Despite its potential benefits, chemotherapy’s broad usage is limited due to unfavorable drug responses, a narrow therapeutic margin, disease tolerance, and inadequate targeting.^5^ Surgical removal of tumors was recommended to be the primary treatment for BC. However, the significant problem is that after surgery, there is a notable risk of cancer reappearing in the same region, which has a negative impact on the outcome of the patient’s medical conditions. To address the issues of conventional therapies, additional treatments such as chemotherapy, radiation therapy, and hormonal therapy are commonly administered alongside surgery to lower the chances of cancer recurrence. Nevertheless, these supplementary treatments also come with the drawback of causing substantial cytotoxicity and adverse reactions in the body.^6,7^

In the past ten years, immunotherapy has become a hopeful substitute for conventional cancer therapies.^8^ Utilizing the immune system offers a promising method for creating personalized and long-lasting treatment strategies. Immunotherapies, which involve activating immune cells with tumor-specific antibodies or inhibiting immune checkpoint pathways, have demonstrated benefits and effectiveness in various clinical trials. Despite these successes, a considerable number of patients exhibit resistance to immunotherapeutic interventions, and the underlying reasons for this resistance remain unclear. Notably, the translation of immunotherapy from preclinical models to clinical applications has led to durable clinical responses in only 20–40% of cases.^9^ Given these factors, there is a pressing need to validate new platforms that can effectively establish anticancer treatments and accurately forecast their efficacy on a single-patient basis.^10^ Therefore, favorable outcomes of the current novel therapies being used in BC rehabilitation have gained more attention than conventional therapies. Indeed over the past few years, enhanced comprehension and utilization of hydrogels have expanded innovative avenues for treating cancer.^11,12^ Among various drug delivery techniques, there has been a notable focus on creating hydrogels using natural and synthetic polymers to act like drug vehicles. These biomaterials offer an intriguing prospect for the development of a scientific model for cancer treatment.^13^

A hydrogel is a structure formed by interconnected polymer molecules that are physically or chemically linked or filled with water-based solutions.^14^ The utilization of hydrogels results in the creation of a controlled drug release system due to their capacity for structural changes, increased porosity to accommodate drug molecules, and their ability to protect drugs from degradation. Moreover, hydrogel is absorbed by the tissue and this interaction helps the drug release in the targeted region and further allows them to remain within the body for extended periods to improve the effectiveness of drug delivery.^15^ Several studies provided proof of the viability and effectiveness of therapy approaches involving hydrogels. These advantages are primarily observed in four key areas: biocompatibility, controllability, high drug-loading ability, and less invasiveness.^16^ A concept of achieving multiple goals with a single approach has emerged: removing tissue initially and promoting tissue regeneration by providing the necessary scaffold or stimulating factors. Hydrogels are well-suited for this task because of their ability to respond to various signals, controlled drug release capabilities, and their capacity to support tissue development. Therefore, neighboring healthy host stem cells can migrate to the damaged area after eliminating abnormal tissue and generate new tissue through growth and specialization.^17^ Wang et al.^18^ proposed a novel technique for local chemo-immunometabolic treatment via supramolecular hydrogel injection. This hydrogel aims to release doxorubicin, which induces immunogenic tumor cell death within the tumor, as well as kynureninase which disrupts the immunosuppressive effects of kynurenine in the tumor region. The combined action enhances the antitumor activity to fight against cancer cells. Recently the *in vivo* mice model study concluded that a low quantity of drug transforms to the tumor region, leading to improved tumor suppression and prolonged mouse survival. From this study, researchers assume that the flexible method of local chemo-immunometabolic therapy can be a universal strategy, enhancing antitumor immunity and amplifying the effectiveness of cancer immunotherapies.^18^ Additionally, thermosensitive hydrogels, a widely explored category among smart hydrogels, have garnered significant attention. Various polymers, including chitosan and hyaluronic acid, demonstrate impressive thermosensitive phase transition characteristics.^19^ Injectable hydrogels, because of their outstanding physical and chemical attributes, hold considerable potential for use in focused diseases and renewed medicine. Additionally, they show promise in addressing conditions such as diabetes, AIDS, and cancer.^20^ Thermosensitive and hydrophilic chitosan was developed to serve as a carrier for locally delivering chemotherapeutic drugs like doxorubicin (Dox), cisplatin (CDDP), or cyclophosphamide (CTX) with the granulocyte-macrophage colony-stimulating factor that acts as an immunomodulator.^21^ At this stage, alginate is extensively employed as a biomaterial due to its cost-effectiveness for large-scale production, minimal immunogenicity, and intrinsic ionic chemical properties, as well as its hydrophilic nature.^22^

This review explores various types of hydrogels and routes through which they can administered for cancer treatment with more focus on sodium alginate and chitosan. Additionally, this review provides an overview of several techniques for creating hydrogels and releasing drugs from them. Furthermore, the study will assess the efficacy and challenges of hydrogels in current biomedical applications and future applications.

## 2 Hydrogel

Hydrogels are prepared by using hydrophilic polymers that form three-dimensional structures. Typically, hydrogels are synthesized by chemically bonding one or more monomers, creating connections between polymer chains. This unique structure allows them to absorb water at their level of hundreds to thousands of times more than their initial dry weight.^23^ Depending upon the synthesis, cross-link methods, and polymer, the hydrogel is differentiated into physical, chemical, and other ways as natural, synthetic, and hybrid.^24−26^ Hydrogels can establish connections through physical or reversible networks, primarily relying on molecular entanglements or various physicochemical interactions like hydrogen bonds, hydrophobic forces, charge interactions, or supramolecular chemistry. These hydrogels are notably adaptable to different environmental conditions due to their strong dependence on external factors such as pH, ionic strength, solvent composition, and temperature. This adaptability differentiates them from materials bonded through covalent bonds.^27^ Chemical cross-linking encompasses two main approaches: one involves attaching monomers onto the polymer’s backbone, while the other employs a cross-linking agent to connect multiple polymer chains. Natural and synthetic polymers be cross-linked by leveraging functional groups like OH, COOH, and NH_2_, which interact with cross-linking agents such as aldehydes or methylene. Various methods detailed in scientific literature exist for creating chemically cross-linked hydrogels that are considered permanent.^28^

### 2.1 Different Types of Hydrogels and their Biomedical Applications

Natural hydrogels are typically thought to be very compatible because of the similarities between their chemical compositions and fiber architectures with the natural extracellular matrix.^29^ Natural hydrogels are primarily based on polysaccharides and polypeptides. In pharmaceuticals, biological fields, and treatments for cancer, natural polymers like hyaluronic acid (HA), alginate, and collagen are frequently employed.^30^ Natural polymers have been investigated for various uses, such as developing tissues, recreating medicine, wound repair, and delivering medicines for cancer treatment. The distinctive characteristics of hydrogels have garnered significant interest, especially in acting as a drug vehicle.^31^ A novel magnetic natural hydrogel was developed and designed to be responsive to pH changes. The hydrogel was composed of alginate, gelatin, and magnetic nanoparticles (Fe_3_O_4_ NPs). The Alg-Gel/Fe_3_O_4_ hydrogel was engineered as a smart drug delivery system (DDS) for cancer chemotherapy. The loaded doxorubicin hydrochloride (Dox) increases the hydrogel’s effectiveness in drug loading. It may also efficiently provide encapsulation and inhibit cervical cancer growth in the Hela cells. Based on the findings, the Alg-Gel/Fe_3_O_4_ magnetic hydrogel can be regarded as an effective and intelligent drug delivery system for cancer treatment and diagnosis.^32^

Musaie et al.^33^ prepared a natural photoactive hydrogel using Bi_2_S_3_ nanorods. It is a photothermal agent coated with hyaluronic acid and loaded in a hydrogel that contains sodium alginate and Farsi gum (FG) coordinated with Fe_^3^_^+^ ions. *In vivo* experiments demonstrated that the hydrogel effectively eliminates tumors through photothermal treatment and speeds wound healing.^33^ Hydrogels containing chitosan act as a potential vehicle for drug delivery systems, making chitosan more attractive due to chitosan’s biological compatibility, high degradability, and low cytotoxicity. Additionally, specialized chitosan hydrogels are prepared efficiently to deliver and target the medicines in response to certain conditions.^34^ The chitosan contains amine (NH_2_) and hydroxyl (OH) groups, due to which it is widely used in organic and inorganic compounds. Terephthaloyl thiourea and chitosan were made of a stable hydrogel, and had homogeneity in aqueous solutions (water), with high permeability.^35^ Rong et al.^36^ prepared a nanocomposite hydrogel that contains hyaluronic acid and chitosan. For better treatment by photothermal, they used mesoporous polydopamine and doxorubicin. The *in vivo* study proved that the complex hydrogel showed extensive cancer treatment.^36^ Hyaluronic acid–dopamine cross-linked with sodium selenite which also contains sodium selenite-mediated hydrogel is mainly used in the case of local therapy of breast cancer.^37^ The extracellular matrix plays a vital role in the development of cancer. The primary element of the ECM, collagens, undergoes significant remodeling in tandem with the emergence of cancer. From various research, it is confirmed that collagen makes a barrier in the development of cancer, so it may be an opportunity to diagnose and treat cancer.^38^ There are two ways to make gelatin hydrogels: photopolymerization and enzyme-mediated cross-linking, which require building covalently interpenetrating networks. Recently Yao et al.^39^ developed a novel gelatin hydrogel using dual enzymatic cross-linking mediated by horseradish peroxidase and galactose oxidase. This innovative hydrogel was employed for incubating mouse bone marrow mesenchymal stem cells, resulting in significantly improved wound healing capacity compared to traditional methods.^39^

Synthetic hydrogel is a type of material prepared through chemical synthesis rather than naturally derived. These hydrogels are typically composed of synthetic polymers or copolymers and can be customized in their physical and chemical properties to align with specific applications. Different hydrogel polymers are frequently employed in biological applications. Some of these include poly(acrylic acid) (PAA), poly(ethylene glycol) (PEG), poly(vinyl alcohol) (PVA), polyacrylamide (PAAm), and polypeptides.^40^ Recently, Choi et al.^41^ prepared a micro-hydrogel using graphene oxide (GO) and poly(vinyl alcohol) (PVA). The prepared hydrogel showed excellent absorption capacity as GO sheets are connected through extra hydrogen bonds, resulting in a transition from a solution to a gel state.^41^ A photopolymerization procedure in the aqueous phase produced artificial hydrogels made of the sodium salt of 2-acrylamido-2-methylpropanesulfonic acid (Na-AMPS). The Na-AMPS hydrogel sheets exhibit favorable characteristics that make them suitable for wound dressings. Notably, they possess high equilibrium water content and excellent oxygen permeability, which are essential for effective wound management.^42^ Kalaithong et al.^43^ designed a polymer hydrogel made of carboxymethyl chitosan (CMCTS) and sodium 2-acrylamido-2-methylpropanesulfonate (Na-AMPS). The study assessed various characteristics of hydrogels, including their gel content, swelling capacity, water retention ability, water vapor transmission rate, and mechanical properties through tensile testing.^43^

Additionally, the research examined the adhesion to skin and cytotoxicity of hydrogel sheets, considering their potential applications in biomedicine, particularly as wound dressings.^43^ Kim et al.^44^ created synthetic ovarian tissue from underdeveloped follicles using a synthetic hydrogel called poly(ethylene glycol) vinyl sulfone (PEG-VS) as a scaffold or framework. The results show that a prepared hydrogel may help to follicles to effectively operate as artificial ovarian tissue for up to 60 days.^44^ Several examples of clinically and experimentally successful injectable hydrogels are reported in Table 1. Recently, there has been a remarkable advancement in preparing and modifying hydrogel-based networks to cater a diverse applications across various fields. The development of novel macromolecular drug delivery systems and biomaterials has evolved astonishingly.

**Table 1:** List of Different Combinations of Alginate and Chitosan Hydrogel for the Treatment of Cancer

Serial no.	Name of hydrogel complex	Loaded molecule	Biomedical applications	Ref.
1	Chitosan/glycerophosphate disodium (GP)	Venlafaxine hydrochloride	Drug release	(45)
2	Carboxymethyl chitosan (CMCS) poly-γ-glutamic acid (γ-PGA) (COP) hydrogel	Oxidized dextran	Wound hemostasis and healing	(46)
3	Chitosan (CS) and galic acid (GA)	–	Tissue engineering and regenerative medicine	(47)
4	Chitosan hydrogel with citric acid (CS-CA-DA)	Dopamine	Repairing spinal cord injury	(48)
5	Poloxamer (PLX)-poly(l-alanine-lysine with pluronic F-127	Tacrolimus	Peripheral nerve recovery	(49)
6	*N*-hydroxysuccinimide (NHS) and 1-ethyl-(3-(dimethylamino)propyl) carbonyl diimide (EDC)	Dopamine	Photodynamic therapy (PDT) and photothermal therapy (PTT)	(50)
7	Chitosan with poly(*N*-isopropylacrylamide)	Ketotifen fumarate	Eye drops	(51)
8	Carboxymethyl chitosan (CMCS) with sodium alginate oxide (OSA)	Curcumin–gelatin nanoparticles	Bacteria-infected wound healing	(52)
9	Hyaluronic acid (HA)	Gallol	Immunotherapeutic	(53)
10	Poly(ethylene glycol) (PEG)	Heparin	Antithrombogenic coatings	(54)
11	Acrylamide (AAm) and *N*-hydroxy methyl acrylamide (HMAm) on β-cyclodextrin (β-CD)	Gentamicin sulfate	Drug release	(55)
12	Polyvinyl alcohol (PVA), corn starch (CS), Castor oil (CO)	Silver nanoparticles of *Mentha piperita* leaves	Wound dressings	(56)
13	Pectin, polyvinylpyrrolidone (PVP), 3-aminopropyl (diethoxy) methyl silane (3-APDEMS), and sepiolite clay	Ceftriaxone sodium	Drug delivery	(57)
14	Poly(ε-caprolactone-*co*-1,4,8-trioxa [4.6] spiro-9-undecanone)– PEG–poly(ε-caprolactone-*co*-1,4,8-trioxa [4.6] spiro-9-undecanone) copolymer (PECT) hydrogel	Cys-Arg-Gly-Asp-Lys modified doxorubicin	Chemotherapy	(58)
15	Cellulose nanocrystals, poly(ε-caprolactone-*co*-lactide-*b*-poly(ethylene glycol)-*b*-poly(ε-caprolactone-*co*-lactide (PCLA)	Doxorubicin	Cancer treatment	(59)
16	mPEG–PLGA	Cisplatin, paclitaxel	Drug release, anticancer activity	(60)
17	Dendritic cells (DC) collagen hydrogel	Doxorubicin/CpG	Chemo-assisted immunotherapy	(61)
18	Methoxy polyethylene glycol (mPEG)	Podophyllotoxin	Cancer treatment	(62)
19	Poly(_D,L_-lactic acid-*co*-glycolic acid)-*b*-poly(ethylene glycol)-*b*-poly(_D,L_-lactic acid-*co*-glycolic acid) (PLGA-PEG-PLGA)	GemC16	Chemo radiotherapy	(63)
20	Alginate (ALG)	Glucose oxidase	Anticancer activity	(64)
21	Chitosan and dextran		Orthopedic implantation surgery	(65)
22	Hyaluronan (HA)	Al_2_O_3_ nanoparticles, multicore magnetic particles (MCPs)	Hyperthermia and bioprinting	(66)
23	Sodium alginate	Axitinib	Immunotherapy	(67)
24	Polyurethane-oxidized dextran (PU-OD)	Doxorubicin hydrochloride	Noninvasive monitoring	(68)
25	Gelatin methacryloyl (GelMA)	Gemcitabine hydrochloride	Osteosarcoma treatment	(69)
26	Cotton-reinforced alginate hydrogel (Alg/CH)	Cotton-reinforced	Liquid absorption	(70)
27	Poly(d,l-lactic acid-co-glycolic acid)-b-poly(ethylene glycol)-b-poly(d,l-lactic acid-co-glycolic acid (PLGA-PEG-PLGA)	Micronized temozolomide	Glioblastoma (GBM) treatment	(71)
28	Dextran–chitosan-based hydrogels	Doxorubicin	Skin cancer therapy	(72)
29	Poly(ethylene glycol)-poly aspartic acid (mPEG-PAsp)	Paclitaxel and cisplatin	Ovarian cancer chemotherapy	(73)
30	Poly(d,l-lactide)-poly(ethylene glycol)-poly(d,l-lactide (PDLLA-PEG-PDLLA: PLEL)	Black phosphorus	Postsurgical treatment of cancer	(74)

## 3 Alginate-Chitosan Hydrogel

Due to distinctive characteristics, (including hydrophilic nature, percentage of water resembling soft tissue, extreme reactivity to biological factors, and sufficient versatility) these materials have become the favored and outstanding option for therapies relating to injury recovery.^75^ Chitosan and alginate are widely used in biomedical applications due to their hydrophilicity and biocompatibility properties.^76^ The mechanical attributes of chitosan can be enhanced through the incorporation of alginate, which offers the added benefits of being biodegradable, biocompatible, capable of absorbing significant amounts of fluids, and, as a polyanionic polymer, able to interact with chitosan’s amino group interacting to its carboxyl group.^77,78^ Chitosan and alginate hydrogels are commonly used in biomedical and tissue engineering applications. This review describe briefly the preparation and properties of alginate-chitosan hydrogel.

### 3.1 Composition and Properties

Chitosan is a linear polymer composed of 2-amino-2-deoxy-d-glucopyranose units linked together through (1 → 4) connections, and it may contain some remaining d-glucosamine units. It can be easily derived through the N-deacetylation process of the chitin heteropolymer, which is highly crystalline.^79^ Several research studies have consistently reported that chitosan possesses two distinct types of reactive functional groups: an amino group is on C2 carbon, and a hydroxyl group is on C3 and C4 carbon. These entities make it easier to alter the physical characteristics of chitosan and produce multifunctional alternatives that can be used for many purposes.^80^ Chitosan is known as a less basic polymer; its p*K*_a_ value is approximately 6.5, which means that its charge density varies within the pH range of 6 to 6.5, and this characteristic gives it pH-responsive properties, which are advantageous for various therapeutic applications. At pH levels below its p*K*_a_, chitosan possesses a high charge density, leading to the formation of polyelectrolytes.

In contrast, at neutral pH, its charge density decreases significantly, resulting in low cytotoxicity and facilitating the intracellular release of biomolecules. Notably, lower charge density also leads to decreased solubility and potential aggregation. Consequently, the type of chitosan used affects the stability of chitosan-based compounds.^81^ Prior research has indicated that chitin can be found abundantly in various natural sources, including the shells of crustaceans like shrimp, crab, and lobster, as well as in fish scales. It is also present in the cell walls of mushrooms, fungi, coral, algae, nematodes, and even in some insects. Chitosan, on the other hand, is derived from chitin through a process known as deacetylation. This transformation involves treating chitin with a high concentration of sodium hydroxide. Furthermore, chitosan can be obtained through alternative extraction methods, including chemical processes, biological approaches, microwave irradiation, and more.^82,83^

Alginate (ALG) is a water-soluble linear polysaccharide made up of irregular blocks consisting of β-d-mannuronic acid (M) and α-l-guluronic residues (G) linked in a 1–4 fashion. This homogeneous (poly-G, poly-M) or heterogeneous (MG) design is determined by how its block-like structure is structured.^84^ Alginate (ALG) is a naturally occurring linear and anionic polysaccharide, that is commonly extracted from the cell walls of brown seaweed species belonging to the Phaeophyceae class. These seaweed species include *Ascophyllum nodosum*, *Laminaria hyperborea*, *Laminaria digitata*, *Laminaria japonica*, and *Microcystis pyrifera*. Additionally, ALG can be synthesized by various bacterial strains, including *acetobacter* and *Pseudomonas* spp. However, for commercial purposes, it is primarily sourced from algae.^85,86^ Although, for the formation of hydrogel commonly cross-linking agents such as N,N-dicyclohexylcarbodiimide, Ca^2+^, and chondroitin could be used.^87,88^ In summary, the versatile properties and intricate composition of this hydrogel represent the unique character of the hydrogel.

### 3.2 Preparation of Alginate-Chitosan Hydrogel

Hydrogel-based sensors have received rigorous demands for mechanical strength, molecular structures, chemical and thermal stability, among other distinctive functions. Advances in hydrogel preparation techniques have enabled the design of sensors with enhanced unique features and structures, aligning with the growing need for increased accuracy and susceptivity.^89^ Alginate and chitosan possess distinct characteristics that render them valuable in diverse applications. Although, the preparation method is crucial in customizing these hydrogels for specific purposes. Researchers have successfully developed various applications, and some of these are briefly described below.

Chitosan-alginate complexes exhibit pH-sensitive properties and have been explored as hydrogels for advancing oral delivery systems for peptides or protein-based drugs.^90^ Interestingly, these polymers are frequently mixed with an additional substance to produce composite hydrogels to enhance their properties. Indeed, a concise example of preparing a chitosan and alginate hydrogel complex was reported by Lin et al.^91^ The chitosan/calcium alginate/bentonite (CTS/CA/BT) composite hydrogel with double-network was synthesized, where one is cross-linked via electrostatic interactions between chitosan and sodium alginate and the other is ionically cross-linked alginate through calcium ions. The combination of bentonite into a double-network polymer backbone can not only improve the mechanical performances of hydrogel but also eliminate the drawbacks of bentonite.^91^ Li et al. developed carboxymethyl chitosan/sodium alginate composite hydrogels for tissue engineering. Three groups were prepared with different amino-to-aldehyde ratios (2:1, 1:1, and 1:2) and further studies were conducted based on its microstructure, physical properties, and cell biocompatibility. The authors investigated that the hydrogel with an amino-to-aldehyde ratio of 1:1 exhibited favorable characteristics, including good porosity, acceptable gelling time, well-built adhesive force, steady swelling rate, and compatibility. This suggests its potential suitability as a scaffold in cartilage tissue engineering.^92^ Also, the study of Afshar et al.^93^ reports a sodium alginate-poly(vinyl alcohol) hydrogel which was developed as a carrier for chitosan nanoparticle drug delivery. Initially, drug-loaded nanoparticles were synthesized using the ionic gelation method. Subsequently, various hydrogel films were prepared with different ratios. The optimal mechanical properties were achieved in the hydrogel film with a 7:3 ratio and 3 wt % of chitosan nanoparticles, as determined by tensile tests. The release profile of the Rosuvastatin drug from the fabricated drug delivery system indicated complete drug release within 24 h, with the chitosan nanoparticles significantly influencing the release behavior.^93^

From several studies, it has been concluded that the synthesized components are meticulously integrated to obtain the required material in the final stage of producing the alginate-chitosan hydrogel. This entails precisely blending alginate and chitosan into two different polymers in precise ratios to create a homogeneous combination. Methods such as ionic gelation, in which oppositely charged ions combine to form a gel, can assist in easing the process. To improve the hydrogel’s physical and chemical properties, researchers carefully considered several factors, such as polymer concentrations and cross-linking agents for a variety of activities.

### 3.3 Biodegradability and Biocompatibility

Chitosan has the potential to significantly influence the growth and defense mechanisms of various plant species. Yet, there remains uncertainty regarding the precise metabolic reactions in plants when exposed to chitosan.^94^ Alginate is widely employed within the food industry for its unique characteristics like stability, thickness, and emulsifying nature. Alginates belong to a category of substances that the FDA generally considers safe. Unlike intravenous forms, oral administration of alginate has not demonstrated significant immune responses. Additionally, it has been reported that often taken orally, alginate shows both nontoxic and biodegradable characteristics.^95^ In reference to antibacterial properties, the positively charged amine (NH_2_) functional group in chitosan can interact with the negatively charged bacterial membrane composed of phospholipids and proteins. This interaction is crucial for the antibacterial effects, particularly in acidic conditions. Consequently, it is necessary to modify chitosan through processes like quaternization and the introduction of cationic groups to enhance this capability under neutral conditions.^96^ Alginate is a naturally occurring polysaccharide known for its exceptional biocompatibility and biodegradability, making it highly versatile in various biomedical applications. Alginate can be quickly processed into different three-dimensional materials, including hydrogels, microspheres, microcapsules, sponges, foams, and fibers, making it a valuable resource in biomedicine.^97^ The occurrence of an immunogenic response at the site of delivery could be attributed to the presence of impurities in alginate, including heavy metals, endotoxins, proteins, and polyphenolic compounds.^98^

According to Karzar Jeddi and Mahkam,^99^ the developed nano carboxymethyl cellulose combined with alginate/chitosan hydrogel has demonstrated outstanding pH-sensitive drug release characteristics in *in vitro* studies. It effectively prevents drug release in the gastrointestinal tract. Notably, the beads exhibit a remarkable pH sensitivity, with the highest drug release occurring at pH 5.8 compared to other pH levels. These findings suggest that hydrogel beads have great potential for drug delivery system.^99^ However, making a hydrogel with alginate and chitosan loaded with LDH/insulin is challenging. Therefore, the ability to promote angiogenesis of core–shell hydrogel beads was assessed through direct contact with the chick embryo chorioallantoic membrane, which demonstrates their biocompatibility and angiogenic potential.^100^ Karim et al. reported that the alginate nanocarriers containing different bioactive compounds could extend their life and simplify the integration of these bioactive compounds into various matrices.^101^ Alsmadi et al.^102^ developed a hydrogel made of alginate and chitosan which was loaded with cisplatin and successfully delivered the drug in the case of lung cancer. The drug reached almost 60% at the target site within 2 h. It was seen that the carrier acts significantly to deliver the drug.

Additionally, its role toward biodegradability and compatibility was also investigated in the case of releasing chitosan on the targeted region of the colon.^102^ Wu et al. prepared a double-layered hydrogel bead composed of chitosan and alginate which demonstrated enhanced mechanical strength, allowing it to resist simulated colon intestinal fluid (SCF), small intestinal fluid (SIF), and gastric fluid (SGF). Drug release from the hydrogel was investigated through *in vitro* processes at various pH values to mimic different physiological conditions. Cytotoxicity was assessed through both *in vitro* and *in vivo* models, revealing that the combination of chitosan and alginate effectively shielded the drug from premature release before reaching the target site. In the *in vivo* investigation, the prepared hydrogel exhibited no toxic effects on normal cells. Conversely, the *in vitro* study indicated that the drug successfully inhibited tumor growth.^103^ Another example Yan et al. (2019) reported that alginate and chitosan have much efficiency of biodegradability and compatibility which were used in the delivery of mRNA vaccine. Both *in vivo* and *in vitro* experiments concluded that the protein loaded with hydrogel showed a five times higher result than the normal one. These findings propose that utilizing injectable scaffold mRNA vaccine delivery could present a promising alternative to conventional nucleic acid immunization approaches.^104^ Furthermore, it is worth noting that alginate and chitosan hydrogel also possess desirable attributes of biodegradability and biocompatibility, making them promising candidates for drug delivery systems with reduced environmental impact and enhanced safety in biological applications.

### 3.4 Current Detection and Diagnosis Techniques Available in Breast Cancer

Breast cancer detection and diagnosis often begins with discussion on your symptoms followed by screening mammography and clinical examination. Several other factors maybe included, i.e., the patient’s age at menstruation, postmenopausal status, past deliveries, and usage of contraceptive pills or following menopause hormone alternatives. Moreover, the medical checkup should be included for a comprehensive visual evaluation with the individual sitting upright.^105^ Indeed, in the case of breast cancer, there are various approaches for the detection of cancer, like clinical screening, imaging, and sometimes collecting the tissue from the patients and analyzing it through biopsy. Another way is biomarkers which are collected from the human body fluid and detected through liquid biopsy.^106^ These imaging techniques mainly include mammography (MG), ultrasonography (US), magnetic resonance imaging (MRI), positron emission computed tomography (PET), computed tomography (CT), and single-photon emission computed tomography (SPECT).^107^

A mammogram is an X-ray of the breast that can reveal benign or malignant abnormalities. It is obtained by applying a small dose of radiation through the breast post-compression between two plates to produce an X-ray image. Mammograms can be utilized for both screening and diagnosis.^108^ Breast MRI has gained recent endorsement from the American Cancer Society as a diagnostic tool for high-risk individuals, including those with a genetic susceptibility or a history of breast elevation during childhood malignity.^109^ Positron emission tomography (PET) is a cutting-edge imaging method involving the injection of a radioactive substance into an arm vein, which accumulates in areas with heightened cellular activity, particularly in cancerous tissue. A specialized PET scanner detects the emitted radiation, producing an image. Combining PET with computed tomography offers a comprehensive physiological and functional perspective of suspect cells. Unlike MRI, factors such as a solid mass on the breast, previous surgery, or radiation have no impact on PET results. Additionally, PET can differentiate benign breast diseases as they appear negative in the scan.^110^ Breast ultrasonography is an affordable and readily accessible screening method that detects tumors by using acoustic waves to interact with breast tissue. To discern the structure of the breast, an ultrasound transducer is utilized to measure the audiovisual waves reflected from the breast. Ultrasonography of the breast is effective in increasing cancer detection rates, particularly in individuals with a high risk of breast cancer. Additionally, it aids in identifying cysts and solid masses.^111^ Once breast cancer is detected by using any one of the techniques which are discussed above, then one needs to take early action for diagnosis. There are many options in the case of diagnosis and therapy of cancer like chemotherapy, gene therapy, photothermal therapy, photodynamic, and radiotherapy. There are various FDA-approved drugs available, and some are in the clinical trial stage for the treatment of breast cancer. Some FDA-approved drugs are Tamoxifen,^112^ Doxorubicin,^113^ Ado-trastuzumab,^114^ Lapatinib,^115^ Tucatinib,^116^ Palbociclib,^117^ and many more. But after all these, there is a need to address the challenges in ongoing research areas to improve health care, awareness, and education about breast cancer to overcome the disadvantages and limitations as discussed in the Introduction.

## 4 Immunotherapy in Breast Cancer

### 4.1 Principles of Immunotherapy

**Figure 1:**
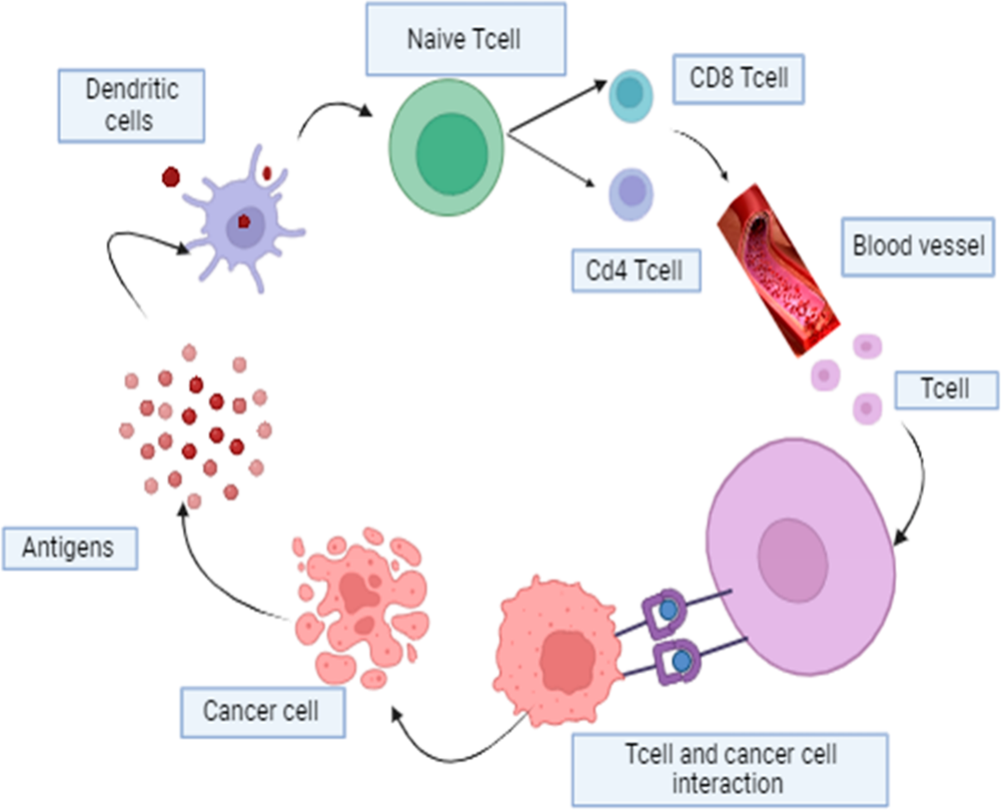
Role of activated T cell in eliminating cancer cells. Dendritic cells (DCs) play a crucial role in activating T cells. When DCs detect antigens from cancer cells, they undergo a process of Naïve T cells possessing and presenting these antigens as peptides on their cell surface through T-cell receptors (TCRs). As a result, the activated T cells eliminate the cancer cells at the tumor site.

The fundamental concept behind cancer immunotherapy is that cancer cells exhibit mutated proteins or overexpress differentiation antigens that antibodies like T-cells can target. The “immunosurveillance theory of cancer” proposed that the immune system could identify and eliminate tumors.^118^ The remarkable achievements in cancer immunotherapy, particularly in treating melanoma, lung cancer, acute lymphoblastic leukemia, and various other cancer types, underscore the potency of T-cell immunity as an external mechanism for suppressing tumors.^119^ Cui et al. represented how the activated cell eliminates the cancer cells as shown in Figure 1.^120^ The examinations of human breast cancer tissue have offered a detailed understanding of the quantity, diversity, and clinical significance of immune cells penetrating breast tumors, as discussed extensively in previous reviews.^121−124^

The cancer immune editing model describes the evolving interaction between the immune system and tumor cells in three pivotal phases.

During the “Elimination” phase, the immune system effectively identifies and eliminates cancer cells. The success of this phase hinges on the immunogenicity of the antigen, which can be summarized as follows: (1) Genetic abnormalities in tumor cells result in the generation of novel antigens. (2) These newly formed antigens are processed and displayed as antigen-derived peptides on the cell surface alongside human leukocyte antigen class I (HLA-I). (3) Neoantigens in the tumor microenvironment are detected, processed, and presented on antigen-presenting cells (APCs) as antigen-derived peptides associated with human leukocyte antigen class II (HLA-II). (4) Helper T-cell receptors recognize these peptides, leading to the stimulation and maturation of B-cells and cytotoxic T-cells. (5) With the assistance of costimulatory signals provided by APCs, activated T-cells identify neoantigens presented by HLA-I, resulting in an attack on the targeted tumor cell. This attack can occur through the secretion of cytotoxic granules and via the engagement of Fas cell surface death receptors (FAS) and caspase activation.

In the “Equilibrium” phase, cells that have undergone transformation and acquired resistant or nonimmunogenic characteristics manage to evade the elimination phase. However, even though these cells continue to increase, the immune system retains some control over the growth of the tumor.

In the “Escape” phase, the selective pressures exerted by anticancer treatments or immune surveillance contribute to the uncontrolled proliferation of cells that exhibit resistance or lack immunogenicity. This unchecked proliferation ultimately drives tumor progression and metastasis.^125,126^ T cells initiate robust and highly targeted immune responses against foreign antigens. Approaches aimed at triggering these immune responses fall under the broad category of T-cell therapies. These therapies are frequently designed with monoclonal antibodies (mAbs) to enhance their effectiveness.^127^

### 4.2 Current Immunotherapeutic Approaches

**Figure 2:**
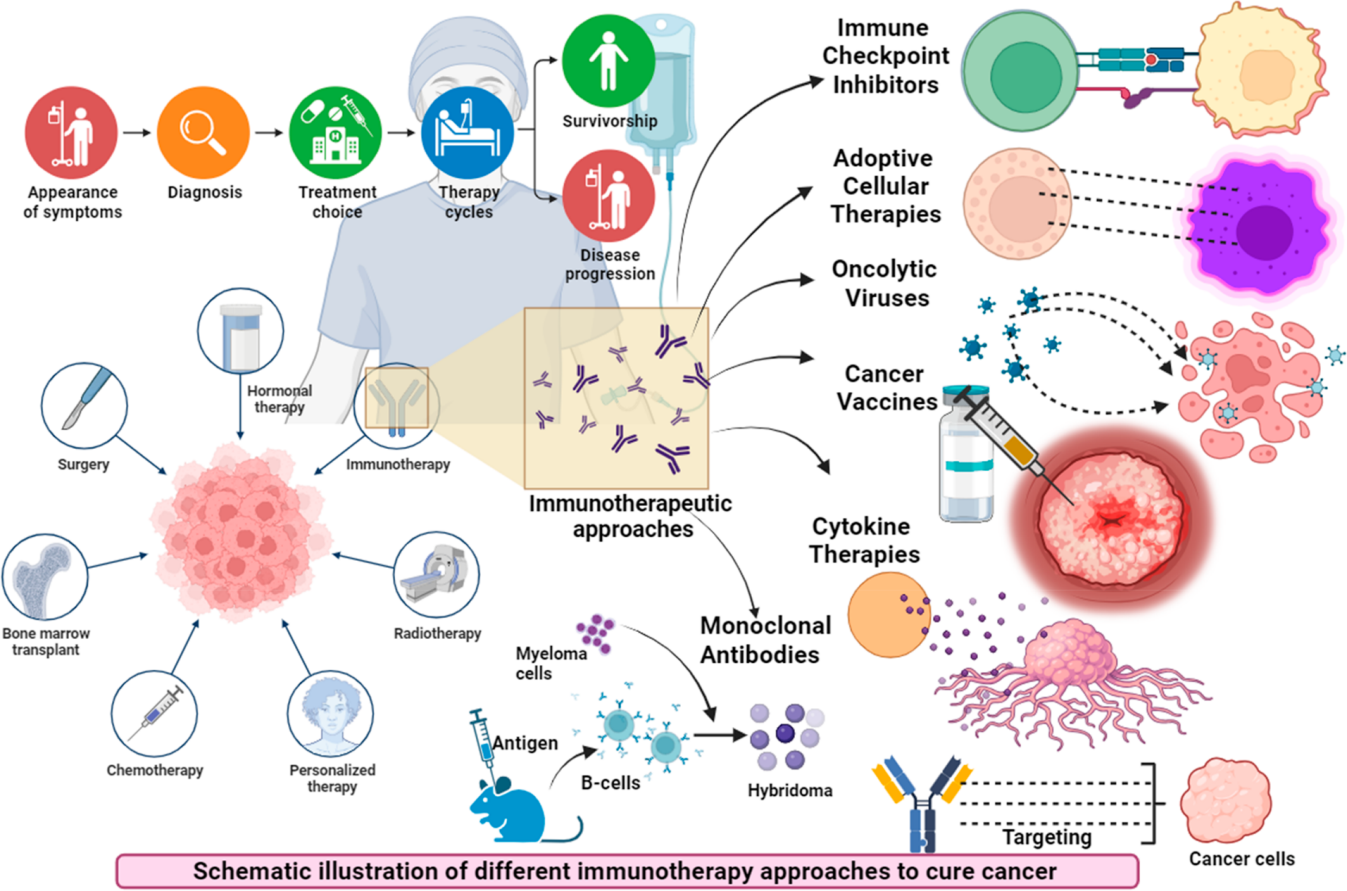
Schematic illustration of different immunotherapy approaches to cure cancer.

The immune system serves a dual role in protecting against tumor development. It accomplishes this by engaging innate and adaptive immune mechanisms while also influencing and molding the immunogenicity of the tumor itself.^128^ Mishra et al. have reported that immunotherapy for cancer includes several approaches as shown in Figure 2, like immune checkpoint inhibitors. These therapies activate T-cell responses by disrupting mechanisms that inhibit them. Adaptive cellular therapies involve introducing engineered immune cells into the body to combat cancer. Oncolytic Viruses: These viruses target and eliminate cancer cells while sparing healthy cells. Cancer Vaccines: These vaccines educate the immune system to recognize and combat cancer. Cytokine Therapies: This approach uses immune-modulating molecules to enhance the body’s immune response against cancer. Monoclonal Antibodies: These engineered antibodies target cancer cells and may stimulate immune responses against cancer.^129^ These diverse strategies represent the spectrum of immunotherapeutic options in cancer treatment.

#### 4.2.1 Immune Checkpoint Inhibitors

T-cells have molecules that can turn off the immune response, preventing an exaggerated response to an infection. However, cancer cells use these checkpoints to avoid being attacked by T cells.^130^ Various immune checkpoint inhibitors include anti-PD-1/PD-L1, CTLA-4, antilymphocyte activation gene 3, and antibodies against T-cell immunoglobulin and mucin-3.^131^ In the 1980s, CTLA-4 and its ligands, B7.1 and B7.2, were discovered on antigen-presenting cells. CTLA-4 was identified in activated effector T cells and immune-suppressive regulatory T cells.^132^ A recent publication delineated a genetic foundation for the clinical response to CTLA-4 blockade in melanoma patients. This advancement enables the differentiation of patients who will respond positively to the treatment from those who will not, even before therapy initiation.^133^ The most extensively researched predictive biomarker for immunotherapy is the PD-1/PD-L1 inhibitory pathway. In certain tumors, PD-L1 expression may be associated with a more favorable clinical response to treatment using anti-PD-1 or anti-PD-L1 antibodies.^134^ Nivolumab, an anti-PD-1 antibody, has shown a notable improvement in overall survival compared to historical control data.

Additionally, antibodies that target PD-L1, such as atezolizumab, durvalumab, and avelumab, have received accelerated approval from the FDA as second-line treatments for metastatic urothelial carcinoma. Remarkably, some patients have experienced durable responses lasting more than a year with these treatments.^135^ The Lag3 marker is another biomarker, that shows a promising alternative target for checkpoint inhibition. In cancer settings, Lag3 is found on activated immune cells and exhausted T cells. Notably, the PD-1 marker with Lag3 is often coexpressed, suggesting that both act as immunotherapeutic agents.^136^ T-cell immunoglobulin and mucin domain-containing molecule 3 (TIM-3) represent a novel immune response regulator associated with T-cell exhaustion. Recent research by Byun et al. has generated significant enthusiasm for exploring immune checkpoint molecules as potential targets in breast cancer therapy, particularly in the context of triple-negative breast cancer (TNBC).^137^

Immune checkpoint inhibitors have emerged as a transformative approach in cancer therapy. They have shown remarkable clinical efficacy across various cancer types, leading to durable responses in a subset of patients. Our understanding of the complex interplay between the immune system and cancer deepens in identifying novel checkpoints like Lag3 and in exploring a set of combined therapies that may open the door for future immunotherapy.

#### 4.2.2 Adaptive Cellular Therapies

Adoptive cell therapy (ACT) represents a potent strategy in which the immune system is customized to effectively target and eliminate tumor cells. This is achieved through the infusion of tumor-infiltrating lymphocytes (TIL) or T cells engineered with novel T cell receptors (TCR) or chimeric antigen receptors (CAR). These innovative treatments have demonstrated encouraging outcomes across a spectrum of tumor types. Moreover, ongoing global clinical trials aim to refine and enhance the effectiveness of this therapeutic approach.^138^ CAR T-cell therapy, TIL therapy (tumor-infiltrating lymphocyte therapy), engineered TCR therapy (engineered T cell receptor therapy), and NK cell therapy (natural killer cell therapy), these four major categories of ACT have shown significant progress in research and clinical applications, offering new avenues for the development of effective and personalized cancer immunotherapies.

#### 4.2.3 Oncolytic Viruses

Due to their unique capabilities, oncolytic viruses (OVs) are gaining prominence in cancer therapy. They present a promising approach by combining targeted destruction of tumor cells with immune system activation, effectively functioning as potential in-body tumor vaccines. OVs can also be genetically modified to improve their selectivity for tumors and enhance their ability to stimulate the immune response. Furthermore, OVs are easily combinable with other treatment agents, making them a versatile tool in cancer treatment.^139^ Lately, the fusion of oncolytic viruses (OVs) with various immunotherapies like immunological checkpoint blockers, chimeric antigenic receptors, target-specific T-cell receptors, and a patient’s tumor-infiltrating lymphocytes (TILs) has shown encouraging advancements in cancer therapy.^140^ Clinical trials have explored the use of oncolytic viruses (OVs) in combination with antibodies targeting various immune checkpoints, with a particular focus on PD-1/PD-L1 and CTLA-4 combination therapies, which have progressed the most.^141^ For example, recently, immune checkpoint inhibitor (ICI) antibodies like Ipilimumab targeting CTLA-4, Nivolumab, and Pembrolizumab targeting PD-1, as well as Atezolizumab targeting PD-L1, have received approval for treating various solid and hematological cancers. Importantly, these therapies have demonstrated sustained clinical responses in some patients, underscoring their effectiveness in cancer treatment.^142^ According to the successful various clinical trials, it has been assumed that oncolytic viruses have emerged as a promising and versatile tool in cancer immunotherapy, offering the potential for targeted tumor cell destruction and immune system activation. Their continued exploration and optimization hold great promise for the future of cancer treatment.

#### 4.2.4 Cancer Vaccines

Cancer vaccines target tumor antigens (TAs) to stimulate cellular and humoral immune responses. These responses work together to impede tumor growth and ultimately eliminate the tumor.^143^ Cancer vaccine platforms are typically classified into three categories: cellular, viral vector, or molecular (DNA, RNA, or peptide) vaccines.^144^ Cancer vaccines can be categorized based on their clinical application into two main types: preventive and therapeutic. Preventive cancer vaccines are developed to induce an immune response to prevent the occurrence of tumors. In contrast, therapeutic cancer vaccines are designed to eliminate existing tumor cells by activating or increasing tumor-specific immune reactions.^145^ The most versatile is the mRNA cancer vaccine, which represents a potent and adaptable form of immunotherapy. As medical trials, particularly those focusing on personalized vaccines, continue to expand, the potential for developing mRNA vaccines tailored to various types of cancer is on the rise.^146^ In addition, therapeutic vaccination to combat cancer has been a research focus for many years. The rapid development and widespread approval of mRNA vaccines against SARS-CoV-2 have showcased the tremendous potential of this technology. The remarkable success of mRNA vaccines in preventing infection has underscored their efficacy. As a response to the COVID-19 pandemic, the development of mRNA-based cancer vaccines has seen enhancements, drawing from years of research data analysis.^147^ Cancer vaccines represent a dynamic and evolving frontier in immunotherapy, offering promise for more effective and personalized cancer treatment strategies in the future.

#### 4.2.5 Cytokine Therapies

Cytokines are small, soluble proteins and are crucial in facilitating cell communication. They can influence the body’s immune response to combat cancer cells and even trigger their programmed cell death. Cytokines were the initial immunotherapy agents to receive FDA approval in late 20^th^ century.^148^ However, research into cytokine-based immunotherapy found more promising for two cytokines, IFN-α and IL-2, which have been granted by the FDA for use in cancer treatment.^149^ Moreover, in hematological malignancies, IFN-2 has recently been re-introduced as a treatment option. It is being used in patients with Philadelphia-negative myeloproliferative neoplasms (MPNs), including essential thrombocytosis, polycythemia vera, and myelofibrosis, as well as in patients with chronic myelogenous leukemia (CML) when combined with tyrosine kinase inhibitors.^150^ The National Cancer Institute conducted Phase II trials with IFN-α in patients with non-Hodgkin lymphoma (NHL). These trials produced mixed outcomes when examining the effects of IFN-α used alone for initial treatment, ongoing maintenance therapy, or when combined with chemotherapy regarding the survival of NHL patients.^151^ TransCon IL-2 β/γ is an innovative prodrug designed for prolonged action, providing a continuous release of an IL-2Rβ/γ-selective IL-2 analogue. The purpose is to overcome the limitations observed in existing IL-2-based cancer immunotherapies like aldesleukin (a synthetic version of IL-2, also known as Proleukin). Adverse events like cytokine release syndrome and vascular leak syndrome are probably a consequence of the frequent administration of high doses, necessitated by the short half-life and the resulting high peak serum concentration of these agents.^152^ Cytokines that promote the upkeep of natural killer (NK) cells, with IL-15 being a prominent example, have been recognized for boosting NK-cell functionality. In experimental mouse models of syngeneic cancers like melanoma, colorectal cancer, lymphoma, and lung cancer, the administration of IL-15 was well-tolerated and supported the proliferation of NK cells. Consequently, IL-15 can serve as a standalone treatment and a supplement to enhance NK cell activity.^153^ Various approaches have been explored to overcome limitations such as toxicity, target limiting, complex mechanisms, and shorter half-life periods. These include attempting to inject cytokines directly into tumor sites and engineering cytokine fusion proteins to augment their antitumor effects.^154,155^

#### 4.2.6 Monoclonal Antibodies

Monoclonal antibodies (mAbs) represent the most frequently employed and approved cancer immunotherapy method in clinical practice. These mAbs are largely made to fight with cancer, specifically tumors of the breast, colon, lymphomas, and other malignancies.^156^ A primary mechanism through which many antibodies induce the death of tumor cells is obstructing the signaling of growth factor receptors. When these antibodies bind to their target growth factor receptors, they disrupt the signaling pathways that promote tumor growth and survival. This can be achieved by altering the activation status of the receptors or by preventing the binding of ligands to them.^157^ Recently, more success has been seen in monoclonal antibodies with different checkpoint inhibitors, for example, a complex made up of mAb with other checkpoint inhibitors such as PD-1 with pembrolizumab, PD-1 with nivolumab, PD-L1 with BMS-936559, and CTLA-4 with tremelimumab. These complex structures offer detailed insights into the antibodies’ specific binding sites (epitopes) and the intricate molecular mechanisms that underlie checkpoint blockade. This information is precious as it helps refine the development of monoclonal antibodies designed to inhibit checkpoint signaling, which is crucial for enhancing the effectiveness of cancer treatment.^158^ Monoclonal antibodies are pivotal in cancer immunotherapy, representing a significant advancement in cancer treatment and a powerful and effective option.

## 5 Role of Hydrogels in Immunotherapy

Hydrogels are immersed in water and make a 3D dimensional structure that is formed from polymers, proteins, small molecules, or colloids. They may play an important role in drug delivery due to their ability to encapsulate drugs, ensuring protection and enabling controlled release over specific spatial and temporal intervals. Therefore, extensive research has been devoted to hydrogels for delivering pharmaceutical products and tiny active chemicals.^159^ The drug delivery depends on load, implantable, injectable, degradable, and stimulus factors.^160^ Hydrogels are well utilized in drug-loaded systems and also this may increasingly be applied in the field of regeneration medicine. Chen et al. presented an example where a hydrogel prepared with calcium alginate has a significant potential for repairing bone defects in clinical applications.^161^ The other characteristic is cross-link, which may stop medicines from spilling out and inhibit the ingestion of multiple proteins that could cause the loaded biological pharmaceuticals to break down.^162^ The interactions between the medicines and polymer chains can be engineered using various methods, including the conjugation process, electrostatic interactions, and hydrophobic associations.^163^ Another one is implanting, during which porous implantable or injectable carriers play a crucial role in enhancing immune cell infiltration into the core of tumor masses. They also stimulate other tumor-specific immune responses, leading to synergistic effects that significantly improve the efficiency of cancer treatments.^164^ Injectable hydrogels can be formulated through *in situ* hydrogel formation, where the hydrogel forms immediately after injection, and injectable microspheres. Implantable hydrogel scaffolds help release cargo, whereas *in situ* hydrogel formation occurs by physical responses like temperature or pH. While injectable hydrogel formulations are preferred for patients due to their minimally invasive nature compared to implantable hydrogels, they come with challenges. *In situ*, hydrogel formulations might be harder to approve because the post-administration of hydrogel products might not form properly or retain complete function, posing potential issues regarding effectiveness and safety.^165^ Simultaneously, cross-linking is a fundamental aspect of hydrogels and is closely linked to various characteristics. Hydrogel networks can be formed through diverse methods, including physical cross-linking through complex aggregation or ionic interaction, chemical cross-linking using cross-linkers, or radiation cross-linking. Each method offers unique properties and advantages, making them suitable for drug delivery.^166^ Recently, Cheng et al. successfully prepared a hydrogel made of sodium alginate, Ca^2+^, which acted as a cross-linker for two targeted drugs, WA-cRGD prodrugs and PD-L1. The hydrogel showed immunomodulatory effects on post-surgery cancer treatment and effectively prevented the recurrence of primary tumors. The positive results imply that the approach has the potential to be a powerful and adaptable treatment method for post-surgery care in a range of solid tumor cases.^167^ Due to the simplicity of delivering anticancer treatments, injectable hydrogels that induce an *in situ* sol–gel transition in the body in retort to chemical or environmental conditions such as pH, temperature, and light following injection are frequently used for local delivery. Indeed, from this perspective, injectable hydrogels serve as highly effective platforms for the local delivery of anticancer agents, ultimately improving the effectiveness of cancer immunotherapy strategies.^168,169^ Biodegradable hydrogels are crucial in innovative disease treatments and novel drug delivery methods. These hydrogels offer versatile solutions, as a range of degradable hydrogel systems can be created by selecting suitable hydrogel precursors or complexes tailored to specific applications. A common two-component system is achieved by hydrogel; this component is composed of both hydrophobic and hydrophilic biodegradable components. The hydrophilic component contributes to the hydrogel’s swelling properties, while the hydrophobic component is responsible for the desired degradation and mechanical features. Importantly, adjusting the ratio of these components allows for precise control over the overall system’s performance, enabling customization based on drug delivery.^170,171^ Hydrogels can respond to specific stimuli, including changes in temperature, pressure, light, magnetic fields, electrical fields, pH, or the concentration of specific molecules in solution. This responsiveness to external factors makes hydrogels valuable in various applications, including drug delivery.^172^ For example, a temperature-responsive nano gel was created to deliver the chemotherapy drug cisplatin to breast cancer cells, which was more effective than the normal case.^173^

**Figure 3:**
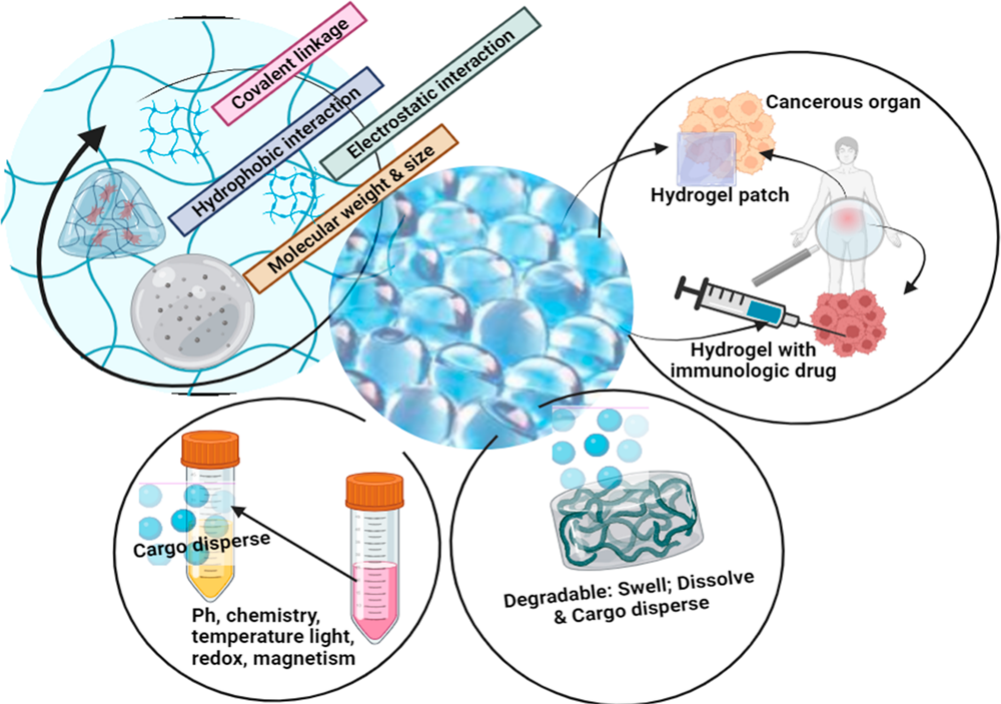
Pictorial representation of different properties of hydrogel to deliver immunotherapeutic drugs.

An additional advantage of employing hydrogels in cancer therapy lies in stimuli-responsive hydrogels. These innovative materials show promising impact and act as smart substances, which might be capable of altering their conformation in response to changes in the neighboring environment, including variations in temperature, pH, light, ionic strength, and magnetic field. The applications and properties of the hydrogel are presented in Figure 3.^174^ This particular hydrogel variety has become notably significant because of its capacity to adjust the rheological characteristics based on diverse conditions within the tumor surroundings. Cancer cells, in their quest for survival, exhibit various metabolic transformations. These include alterations in glucose and nutrient uptake, the generation of lactic acid even in aerobic conditions, and adjustment to hypoxic and low-nutrient microenvironments. Additionally, cancer cells undergo extracellular acidification, leading to low pH, while the cytoplasm experiences intracellular alkalization, resulting in high pH.^175^ These distinctive characteristics offer an opportunity to exploit them in the development of stimuli-responsive drug delivery systems targeted specifically at tumors, showcasing an advantage in utilizing hydrogels for this purpose.^176^

### 5.1 Alginate-Chitosan Hydrogels in Breast Cancer Immunotherapy

Breast cancer is the most common cancer, the most common cause of death in women due to its motility and morbidity rate. The survival rate is less than 30% after adjuvant chemotherapy.^177^ Neoadjuvant therapy now acts as an option for scientists to find out the best result for the treatment of breast cancer. Standard treatments like surgery and radiation therapy are the most known post-surgery therapies; although, adjuvant systemic therapies are given to eradicate potential late-stage cancer.^178^ Chemotherapy, although effective, sometimes does not work as selectively and acts as toxicity upon healthy cells; for example, patients have suffered from loss of appetite and vomiting. It also shows that it works in high doses, making it more toxic to normal cells and resistant to the drug. Indeed, these harmful effects on healthy tissues and organs play a significant role in the elevated death rates among patients.^179^

Immunotherapy has become a hopeful approach to cancer treatment, generating long-lasting immune responses in patients with malignant tumors. The primary goals of cancer immunotherapy include boosting the capabilities of antigen-presenting cells, encouraging the generation of defensive T-cells, and getting control over immunosuppression.^180^ Inoculation of biodegradable hydrogels is an excellent option for localized drug release due to several advantages. This may allow higher drug dosages at the tumor site compared to systemic delivery, reduce unfavorable effects on normal tissues, ensure sustained and controlled drug release, and enable the incorporation of multiple synergistic drugs within the same hydrogel for merger therapy.^181−183^ Preclinical models, such as genetically engineered mouse models, patient-derived xenografts, and two- and three-dimensional cell cultures, have become valuable tools for investigating the implementation of cancer development and assessing the effectiveness of anticancer drugs.^184^ Here we have discussed the role of alginate and chitosan hydrogel in the treatment of breast cancer in the various preclinical models.

Alginate hydrogels possess remarkable properties with less toxic, and highly steady factors, along with greater biocompatibility and biodegradability features. These features make them ideal candidates for taking drugs, primarily low molecular drugs and macromolecules such as proteins and genes, in a sustained or localized manner.^185^ There are some recent developments in immunotherapy treatment against breast cancer. A group of researchers formulated alginate hydrogel biopolymers (gelatin and alginate) to develop a hydrogel capable of encapsulating living HER2+ breast cancer cells BT-474/GFP. The hydrogel could enter the cells, which is evaluated by physicochemical characterization. The created hydrogel exhibited favorable characteristics, swelling by 38% of its original mass within 20 h and having pore sizes ranging from 20 to 125 μm. These properties enabled cells to retain their structure in a 3D environment. Additionally, the hydrogel demonstrated biocompatibility, maintaining 90% of cell viability over 10 days. Moreover, the encapsulated BT-474/GFP cells retained their HER2 expression, detectable using the Trastuzumab-fluorescent antibody. Hence, this hydrogel holds the potential for evaluating novel HER2-targeted therapies.^186^

To trigger a specific immune response against cancer self-antigens, Hwang and co-workers developed an injectable hydrogel called thermally responsive hydrogel (pTRG) using alginate-collagen. This hydrogel was infused with indocyanine green and the immune stimulator polyinosinic: polycytidylic acid (poly I: C). They tested pTRG’s effectiveness against CT-26 carcinoma and 4T1 breast tumors in mice by combining photothermal therapy (PTT) and immunotherapy. Near-infrared (NIR) irradiation raised the temperature in pTRG, leading to therapeutic effects in mouse tumors. In mice injected with CT-26 tumors, pTRG treatment prevented lung metastasis through cancer individual antigen T cell immunity. Furthermore, pTRG successfully eradicated original tumors in 4T1 tumor-bearing mice using PTT and shielded them from lung metastasis. We also incorporated various immunotherapeutic molecules into TRGs, eliminating initial CT-26 tumors and preventing lung metastasis. The findings emphasize that TRG is highly effective in treating primary tumors and preventing both development and reappearance.^187^ Wang et al. demonstrated that injecting macroporous alginate gels around the tumor site (loaded with granulocyte-macrophage colony-stimulating factor (GM-CSF) to concentrate dendritic cells (DCs), CpG oligonucleotides, and a doxorubicin-iRGD conjugate) enhances the death of tumor cells by immune responses. This approach boosts the presence of systemic tumor-specific CD8+ T cells and transforms tumor-connected macrophages into an inflammatory M1-like phenotype. It also crucially enhances the effectiveness against triple-negative breast cancers (TNBCs), which have less immunogenicity. Moreover, it stops tumor repetition postsurgical removal. This chemo-immunotherapy method, which concentrates DCs to present naturally occurring tumor antigens in the body, holds promise as a straightforward platform for altering the suppressive tumor microenvironment.^188^ In a study, the researchers aimed to develop a novel drug with the help of hydroxyapatite (HAp) polymer and sodium alginate (NaAlg), which encapsulated iron(III) oxide nanoparticles. The drug was characterized by using various techniques. The synthesized compound was spherical in the range between 9 and 13 nm. In the acidic condition (pH 5.5), the drug’s loading was higher than the standard case. The study found that catechin hydrate-coated iron oxide nanoparticles showed increased toxicity against HT-29 and MCF-7 cancer cells. The *in vitro* study suggests that the prepared compound can inhibit the growth of tumors in the case of breast cancer.^189^

Another study reported against the MDA-MB-231 and MCF-7 breast cancer cells, where the researchers prepared hydrogel folic acid (FA)-grafted chitosan-alginate nanocapsules (CS-Alg-NCs) loaded with turmeric oil (TO). By the characterization, it concluded that it has size, i.e., 189 nm, with favorable drug loading capacity and encapsulation activity. With a comprising study, it was found that TO-FA-CS-Alg-NCs have lower cell viability activity against the breast cancer cell lines. This finding indicates that the hydrogel improves the anticancer effectiveness in the case of breast cancers with high folate receptor (FR) expression.^190^

Chitosan, a β (1–4) glycan resulting from chitin N-deacetylation has accumulated attention in drug delivery due to its capacity to regulate immunological reactions and biological compatibility. It has received approval from the FDA for applications in the food and pharmaceutical industries.^191^ Chitosan also shows its medico-properties on various breast cancer cell lines. Li et al.^192^ formulated a hydrogel made of thiolated chitosan (CSSH), loaded with doxorubicin (DOX) and halloysite nanotubes (HNTs). The study demonstrated that HNTs-SH could be uniformly distributed in the gel, increasing its compression resistance. It was found that in the MCF-7 cell line the effective release of the drug DOX from the hydrogel and after tumor removal effectively suppressed the return of cancer and repaired the damaged tissue. Hence, as a result, it concluded that the prepared hydrogel could inhibit the growth of tumors in breast cancer.^192^ Chen et al.^193^ developed an innovative immunotherapy approach utilizing injectable reactive oxygen species (ROS) within responsive hydrogels. These hydrogels sustainably release 5,6-dimethylxanthenone-4-acetic acid (DMXAA), a STING agonist, and indocyanine green (ICG) in response to the high ROS levels in the tumor microenvironment (TME). Combining the STING agonist with photothermal therapy (PTT) enhances the effectiveness of DMXAA. It may also be transforming the immunosuppressive TME into an immunogenic and tumoricidal microenvironment, leading to complete tumor cell eradication. This bioresponsive gel may harness local ROS to facilitate immunotherapy drug release. Thereby improving combination therapy efficacy, modifying the TME, restraining tumor growth, inducing memory immunity, and protecting against tumor rechallenge.^193^ In this study, a novel approach for breast cancer treatment has been developed involving gefitinib-loaded cellulose acetate butyrate nanoparticles (Gnb-NPs) loaded in chitosan/β-glycerophosphate hydrogels. The optimized Gnb-NPs were then incorporated into chitosan hydrogels by forming Gnb-NPs-hydrogel. This formulation exhibited desirable characteristics, including spherical particles with a size of 156.50 ± 2.40 nm, high encapsulation efficiency, and controlled drug release rate. Gnb-NPs-hydrogel demonstrated superior cytotoxicity against 4T1 breast cancer cells compared to free gefitinib and gefitinib-loaded hydrogel. *In vivo*, studies further confirmed the robust antitumor efficacy of intratumorally injected Gnb-NPs-hydrogel in mice with breast tumors. These results underscore the potential of Gnb-NPs-hydrogel as a promising treatment strategy for breast cancer.^194^

Monette et al.^195^ have enhanced the immunotherapies in case of breast cancer by delivering tumor-specific T-lymphocytes coated with chitosan. For the ideal cell encapsulation, chitosan has been found best for its biocompatibility and thermostability with excellent mechanical properties and cytocompatibility. The CTGel2 formation demonstrated superior performance compared to others and created an environment conducive to the enclosing of viable CD8+ T lymphocytes. This formulation supported the increase and continuous release of T cells. Also, their phenotypes were influenced by the surrounding conditions while preserving their ability to kill tumor cells. These findings strongly indicate that cells enclosed in this formulation maintain their anticancer functions. Therefore, this injectable hydrogel holds promise for further development as a complementary approach to various immunotherapies.^195^ Alioghli Ziaei et al.^196^ have formulated a nanogel made of oxidized alginate and gelatin-containing doxorubicin (DOX)-loaded chitosan/gold nanoparticles (CS/AuNPs). The standalone use of these nanoparticles (NPs) in drug delivery encounters limitations, particularly regarding proteins binding to their surface in serum, resulting in decreased performance. Chitosan (CS), being a biodegradable and biocompatible natural polymer, offers a solution by not only covering the surface of gold nanoparticles (AuNPs) but also facilitating their additional formation method. Interestingly, when the hydrogel containing the drug and free DOX at the same concentration was applied, then it may led to a notable increase in MCF-7 cell death, showcasing the potential of the developed hydrogels for localized breast cancer treatment.^196^

Polymeric nanoparticles consist of biocompatible or natural polymers, including poly(lactide-*co*-glycolide), poly(ε-caprolactone), chitosan, alginate, and albumin. Certain formulations, including Abraxane and Ontak, have received approval from the FDA.^197^ Shen et al. have reported that alginate and chitosan can drug deliver in breast cancer by using *in vivo* cancer models. Here the anticancer drug doxorubicin was loaded in BSA gel, where the BSA gel capsule was composed of a chitosan–alginate capsule wall and BSA gel core. They created a tumor model using nude mice that carried MCF-7/ADR tumors. After the treatment of the prepared gel, the weight of the tumor gradually decreases. The successful loading and prolonged release form the basis for utilizing BSA-gel-capsules in cancer treatment. Additionally, the outcomes highlight the potential use of polyelectrolyte microcapsules, particularly in drug delivery applications for localized chemotherapy.^198^ Miranda et al. reported breast cancer by treating it with glycoalkaloid extract to determine its cytotoxicity as well as the chemosensitizing effect of cisplatin. They tested with RT4 cells and PDX cells. They used alginate/gelatin hydrogel to grow the tumor cells in a 3D bioprinting model. In both RT4 cells and PDX cells, the IC50 values were 2.16 times and 1.4 times higher, respectively, in three-dimensional cultures compared to two-dimensional monolayers. In summary, the study showed the cytotoxicity effect of GE on BC cells and also demonstrated that GE could sensitize BC cells to chemotherapy.^199^

The United States Food and Drug Administration (FDA) oversees the drug acceptance process and assesses new medical devices and drugs before their market release. While the FDA itself does not conduct drug testing. It plays a crucial role in extensive research focusing on the quality control, welfare, and potency of drugs. Clinical trials represent a significant and conclusive phase in the approval processes, also serving as a pivotal step before meeting all FDA requirements and making new drugs available to consumers who require them. In the National Library of Medicine, it is reported that chitosan has been used Interventional in Breast Cancer Surgery: Axillary Dissection Condition and the other one in Breast Cancer Stage IIIA, Breast Cancer Stage IIIB, and Breast Cancer Stage IV conditions, but both are phase 3.^200^ Along with these, here are several other examples of success in treating breast cancer through hydrogel presented in (Table 2).

**Table 2:** List of Chitosan and Alginate-Based Drug Delivery Systems for Breast Cancer Treatments

Serial no.	Hydrogel composition	Drug/agent	Breast cancer cell line as a model	Application/therapeutics	Ref.
1	Alginate-polydopamine (Alg-PDA)	Dopamine	4T1	Photothermal therapy	(201)
2	Chitosan (CS)-agarose (AG)-montmorillonite (MMT)	Curcumin	MCF-7	Cytotoxicity activity	(202)
3	Gel/PDMAEMA/PNIPAAm/Fe3O4 magnetic hydrogel (MH1, MH2)	Doxorubicin hydrochloride	MCF-7	Hyperthermia therapy and chemotherapy	(203)
4	Hyaluronic acid (HA)	Paclitaxel (PTX) nanoparticles and epirubicin (EPB)	MCF-7	Prevent recurrence and distant metastasis	(204)
5	Chitosan hydrogel	5-Fluorouracil	MCF-7	Drug release	(205)
6	Collagen and polyvinyl alcohol (PVA)	Paclitaxel-nanoparticles	MCF-7	Local treatment after surgical resection	(206)
7	Chondroitin sulfate multi aldehyde (CSMA), branched polyethylenimine (BPEI) and BPEI conjugated graphene (CSMA/BPEI/BPEI-GO)	Doxorubicin	MCF-7	Postoperative recurrence prevention	(207)
8	Polydopamine with thiolated hyaluronic acid(PDA- HA-SH)	Doxorubicin	4T1	Chemo-photothermal immunotherapy	(208)
9	Pluronic F_127_ hydrogel	Ti_3_C_2_ nanoparticles	4T1	Photothermal therapy	(209)
10	Chitosan-based hydrogel	Resveratrol, DOPA-rGO	MCF-7	Chemo-photothermal therapy	(210)
11	PDA–PAM hydrogels	BPD-BBTD-NPs	MCF-7	Photothermal therapy	(211)
12	Graphene oxide with folic acid- hyaluronic acid-chitosan-*g*-poly(*N*-isopropylacrylamide) GOFA- HACPN	Doxorubicin	MCF-7	Intratumoral drug delivery	(212)
13	PCNA-GNRs	Doxorubicin	4T1	Preventing postoperation cancer relapse	(213)
14	Alginate-conjugated trimethyl chitosan nanoparticles (ATMC NPs)	siRNA	4T1	Inhibition of S1PR1 and GP130	(214)
15	Capsaicin-loaded alginate nanoparticles in polycaprolactone-chitosan nanofibers (Cap-ALG NPs in PCL-CS NFs)	Capsaicin	MCF-7	Prevention and treatment of cancer	(215)
16	L+GC+ICG, GC@ICG	–	4T1	Treatment of cancer	(216)
17	d-α-tocopherol polyethylene glycol 1000 succinate conjugated chitosan (TPGS-*g*-chitosan NP)	Docetaxel	SK-BR-3	Breast cancer therapy	(217)
18	TAT peptide- hyaluronic acid-trimethyl chitosan-thiolated chitosan nanoparticles (HA-TAT-TMC-TC NPs)	siRNA	4T1	Target PD-L1 and STAT3	(218)
19	Cinnamomum cassia essential oil with chitosan nanoparticles (CS-CEO NPs)	–	4T1	Target caspase-3 and AIF	(219)
20	Chitosan/alginate nanoparticles	Antisense oligonucleotides	T47D	Reduce the expression of EGFR	(220)

## 6 Efficacy and Challenges

Immunotherapy in the case of breast cancer shows success in clinical trials, for example, blocking the checkpoints, and increasing the activity of T cells.^221^ By combining immunotherapeutic agents with hydrogels, studies aim to enhance targeted delivery and sustained release of immune stimulants, effectively modulating the immune response against breast cancer cells. This approach can improve treatment outcomes by maximizing the therapeutic impact of immunotherapy while minimizing adverse effects on healthy tissues. There is a growing interest in utilizing chitosan-based drug carriers for breast cancer (BC) treatment. In the case of clinical application, the formulated chitosan nanoparticles have gained significant attention. These nanoparticles are versatile drug carriers with excellent biocompatibility and are easily modifiable for specific therapeutic purposes.^222^ The ability of chitosan to stop the activity of M1 macrophages and change the surrounding environment of the tumor enhances the effectiveness of cancer immunotherapy.^223^ Alginate has gained considerable attention as a potential carrier for delivering variable molecular weight of drugs. Its applications in pharmaceutical and biomedical research were found very promising. Critical attributes of alginate, such as its safety, biocompatibility, and straightforward preparation methods, underscore its significance.^224^

In some cases, it has been seen that alginate acts as an insufficient attachment in cells, degradation, and burst release. However, alginate has some drawbacks, such as its poor cell adhesion, lack of mechanical and hydrophilicity properties. To overcome these drawbacks, the researchers have taken a challenge that has been trying to combine the compound with various other natural or artificially prepared compounds.^225^ For example, versatile carriers were created by combining alginate, gold nanorods, and superparamagnetic iron oxide nanoparticles for therapeutic research. These carriers were engineered for precise and regulated release of doxorubicin upon exposure to a near-infrared laser while enabling imaging. The nanosystems were designed to be easily tracked using magnetic resonance imaging in the T2 imaging mode.^226^ Like this, the alginate is combined with distinct compounds loaded with anticancer drugs like paclitaxel, doxorubicin, tamoxifen, curcumin, and various others for the eradication of breast cancer.^227^ From the above discussions, it may be concluded that the alginate-chitosan is suitable for targeted drug delivery, immunomodulation, therapeutic synergy, and diagnostic applications. Moreover, some challenges were also seen in a few cases, like optimal standardization of the dose of anticancer drugs and variation in the impact of hydrogel’s efficacy on patients. It might be a challenge for researchers to find success at the clinical level and face regulatory obstacles and scaling problems.

## 7 Conclusion and Future Outlooks

Breast cancer remains a formidable challenge in oncology, demanding innovative and targeted therapeutic approaches. Conventional treatments like chemotherapy, hormone therapy, and immunotherapy alone have shown varying degrees of success, but they often come with significant side effects due to their nonspecific nature. Hydrogel-based immunotherapy offers a promising solution to these challenges for several reasons and those are targeted therapy, sustained drug release, enhanced immune response, reduced systemic toxicity, and overcoming drug resistance. In summary, hydrogel-based immunotherapy for breast cancer represents a promising route for treatment. Researchers have extensively investigated the two versatile hydrogels, alginate and chitosan, to discover their unique properties and multifunctionality, particularly in targeted cancer therapy and drug delivery. These may show stability and change the tumor environment by increasing the immune response. While some drug carriers have faced challenges in clinical translation, however, ongoing research into hydrogel-based formulations shows promising results. These advancements signify a potential shift toward new and innovative modalities for breast cancer treatment in the coming years. For future investigations, there is a need to optimize the ratio of hydrogels to get more stability in releasing the drugs and break down the required drug on the targeted site clinical trials.

Additionally, combination therapy (i.e., therapy combining hydrogels with various cell types for example, stem cells) should be kept as a focus to improve the hydrogel’s performance in patients. *In vivo* model is crucial for studying cancer biology and testing drugs, but they often fall short of accurately mirroring the clinical scenario due to the absence of either human cells or a functional immune system. Along with clinical studies, failures are often encountered due to challenges in replicating original laboratory conditions, issues with scalability, and complex experimental designs that hinder the production of drugs. Quality control standards may not be met, and inconclusive results from animal tests to trials in a limited number of patients contribute to setbacks. Emphasizing the advancement of computational models, along with increasing the trials in animals and humans under diverse conditions, holds the potential to yield more comprehensive information and definitive results during the approval stages of new delivery systems. This underscores the need for a heightened focus on clinical trials to enhance the reliability and success of drug development. Also, the advanced imaging techniques may play a very important role in monitoring hydrogel drug release, simultaneously finding drug release and treatment response, providing a platform for individualized treatment approaches. The researchers should pay attention to preclinical and clinical studies to confirm the hydrogel’s effectiveness and safety in human participants. Summarizing conclusions and outlining these future outlooks must provide a roadmap for researchers and clinicians, guiding them toward a promising route in the continued exploration and application of Alginate-Chitosan hydrogel for breast cancer immunotherapy and diagnosis.
